# Cardiac Arrest Due to Perioperative Anaphylactic Shock Induced by Re-Exposure to Propofol: A Case Report

**DOI:** 10.3390/jcm13185548

**Published:** 2024-09-19

**Authors:** Jin Joo, Hyun Jung Koh

**Affiliations:** Department of Anesthesiology and Pain Medicine, Seoul St. Mary’s Hospital, College of Medicine, The Catholic University of Korea, Seoul 06591, Republic of Korea

**Keywords:** anaphylaxis, anaphylactic shock, cardiac arrest, propofol, tryptase, skin test

## Abstract

Anaphylaxis is a potentially life-threatening systemic allergic reaction that can result in fatal outcomes if not promptly and appropriately treated. The diagnosis of the cause of anaphylaxis during anesthesia can be challenging due to the complexity of the perioperative environment. Propofol-induced perioperative anaphylaxis is uncommon, occurring in perioperative anaphylactic shock cases. We present a case of perioperative anaphylactic shock in a patient with no known allergies who had been exposed to the same anesthetic agents, propofol, rocuronium, and remifentanil, three times previously without incident. Cardiac arrest occurred 50 min after induction, which showed pulseless electrical activity with decreasing saturation without bronchial spasm and skin erythema or edema. After prompt and appropriate management including cardiopulmonary resuscitation, the patient recovered without complications. The diagnosis was confirmed as propofol-induced anaphylactic shock by an elevated serum tryptase level, measured in a timely manner, and by skin tests (skin prick test and intradermal test), which revealed strong hypersensitivity to propofol. This case is notable for the cardiovascular collapse that occurred without respiratory symptoms or skin manifestations, as well as the delayed onset of anaphylaxis (>50 min). This case underscores the importance of vigilance for anaphylaxis, even with repeated exposure to previously well-tolerated drugs, as sensitization can lead to more severe reactions. It also highlights the potential for anaphylaxis to occur outside the acute phase and without typical clinical features.

## 1. Introduction

Anaphylactic shock is a rare but serious and potentially fatal systemic allergic reaction that requires immediate treatment. The incidence of anaphylaxis during general anesthesia has been reported in the range of 1 in 10,000–20,000 cases [1,2]. It typically presents with cardiovascular collapse, respiratory distress due to airway spasm, and skin manifestations such as flushing and/or edema. During an anaphylactic reaction in the perioperative setting, the identification of the causative agent among the various administered medications is often challenging.

Neuromuscular blocking agents (NMBAs) are the most common cause of anaphylactic shock during anesthesia, contributing to 60–70% of cases. Other potential triggers include antibiotics, particularly penicillins, cephalosporins, and other beta-lactam antibiotics, as well as intravenous anesthetic agents (e.g., barbiturates). Among barbiturates, thiopental has the highest incidence rate at ~1 in 30,000; thiopental-related occurrence is more likely in patients with prior exposure and in women [3].

Propofol (2,6-diisopropylphenol) is an intravenous anesthetic formulated in a lipid vehicle containing soybean oil, egg lecithin, and glycerol. Most cases of propofol-induced anaphylaxis occur upon first exposure during the induction of anesthesia. Propofol may directly stimulate histamine release, an effect that may be exacerbated when administered alongside muscle relaxants. Patients with a history of exposure to histamine-releasing muscle relaxants or several proven drug allergies are at greater risk of experiencing anaphylactic shock and advised against using propofol [4]. Although rare, perioperative anaphylaxis can be presented as a serious reaction caused by propofol contributed to trigger with opioids such as fentanyl synergically [5]. However, opioid- or propofol-induced anaphylaxis is less common than expected.

In this report, we present a case of unexpected cardiac arrest due to anaphylactic shock following exposure to propofol.

## 2. Case Presentation

A 59-year-old man (165 cm, 72 kg) was scheduled to undergo arthroscopic exploration with ligament reconstruction due to left medial talar osteochondritis dissecans (OCD). His medical history included diabetes, hypertension (HTN), and dyslipidemia. Preoperative evaluation showed bradycardia (48 beats/min, bpm) on ECG reading without other abnormalities. The patient had no history of allergic drug reactions (ADRs). He had previously undergone three surgeries: arthroscopic surgery on the left knee three years prior, arthroscopic ligament repair of the right shoulder 2 years prior, and spinal surgery (decompression at L4-5, right microdiscectomy at L5-S1) 3 months prior. During all previous surgeries, anesthesia was administered using the same drugs (propofol 1.5 mg/kg, remifentanil 0.1–0.2 μg/kg/min, and rocuronium 0.6–0.8 mg/kg) without any complications.

Before the current operation, the patient was referred to a cardiologist. Despite the presence of HTN and bradycardia, the operative risk was considered low because HTN was well controlled and bradycardia was maintained without heart block. An antibiotic skin test (AST) which is routinely performed preoperatively was not performed because a prior AST for cefazolin 3 months earlier had been negative. As in previous surgeries, the patient was administered 1% propofol (100 mg intravenously) and rocuronium (50 mg intravenously), followed by endotracheal intubation. Hemodynamic stability was maintained at the heart rate (HR) of 50 to 60 bpm with sevoflurane 1.8 vol% and remifentanil 0.1–0.2 mg/h.

After the patient had been positioned in the left lateral decubitus position, the surgical team conducted draping and preparation for surgery. Cefazolin 1 g was administered intravenously ~30 min after induction. Approximately 50 min later, peripheral oxygen saturation (SpO_2_) suddenly decreased from 97% to 90%. End-tidal CO_2_ (EtCO_2_) did not decrease, and airway pressure remained within the normal range (16–18 mmHg). Manual ventilation confirmed no ventilator malfunctions, and lung auscultation revealed no wheezing or high airway pressure. Despite these checks, SpO_2_ continued to decline without recovery, and femoral artery pulsation became undetectable. At this point, blood pressure (BP) was 35/27 mmHg and HR was 50 bpm.

The patient was presumed to be experiencing cardiac arrest with pulseless electrical activity (PEA); thus, cardiopulmonary resuscitation (CPR) was initiated with chest compressions and administration of epinephrine 1 mg. The return of spontaneous circulation (ROSC) was achieved within 2 min. Invasive hemodynamic monitoring, including arterial cannulation and central line insertion, was initiated. Vasopressors, such as norepinephrine (0.02–0.05 mg/kg/min) and vasopressin (1.5–3 U/h), were infused with epinephrine 100 mcg bolus intermittently until systolic BP > 100 mmHg. Transesophageal echocardiography (TEE) detected no abnormalities in wall motion or volume state, and cardiac markers (Troponin-T, CK-MB, NT-pro BNP) were checked.

After CPR, the patient’s ECG showed ventricular premature complexes (VPC) in bigeminy. Despite this, other vital signs, including BP, SpO_2_, and HR, returned to normal and stabilized. The operation was hold without further progress, and the patient was transferred to the surgical intensive care unit (SICU) for further evaluation and management. The following day, he was moved to a ward without any new abnormal findings. A transthoracic echocardiogram (TTE) showed a left ventricular ejection fraction (LVEF) of 64.9%, normal left ventricular systolic function, frequent VPCs, mild left ventricular hypertrophy (LVH), and mild to moderate aortic regurgitation (AR). The cardiac markers showed no specific abnormalities, except for mildly elevated Troponin-T (Table 1). The patient was discharged a few days later without complications.

Based on the findings, anaphylactic shock was considered a likely diagnosis, though initially difficult to identify due to the absence of clear respiratory and skin symptoms. To investigate this, serum tryptase tests were conducted at the time of the cardiac arrest, 1 h later, and 8 h later. The tryptase level peaked 1 h after arrest and then gradually decreased, supporting anaphylaxis as the primary cause (Table 2).

Eight weeks later, an allergist conducted skin tests in two stages: a skin prick test and an intradermal test. The tested agents included all drugs administered prior to the anaphylactic shock: propofol, rocuronium, cephalosporin, and fentanyl citrate, substituting remifentanil. The results were positive for propofol, marginally positive for fentanyl, and negative for rocuronium (Table 3). Consequently, these findings were documented as an adverse drug reaction (ADR), and a drug alert card was issued to prevent future incidents.

## 3. Discussion

Anaphylaxis is a severe, life-threatening allergic reaction that manifests as a generalized or systemic hypersensitive reaction. It is typically an immunoglobulin E (IgE)-mediated hypersensitivity reaction (type I), characterized by rapidly developing problems involving the respiratory system (airway, breathing), cardiovascular system (hypotension, tachycardia), and circulation, with associated skin and mucosal changes. Sometimes, the symptoms of anaphylaxis may be mistaken for those of an acute cardiovascular or respiratory event. Therefore, measurements of serum tryptase are crucial to determine whether anaphylaxis is the cause. The timing of tryptase measurement is critical; an acute elevation indicates mast cell degranulation, and the measurement should be performed within 1–2 h after the onset of the reaction to confirm mast cell activation [6].

In Korea, most cases of drug-induced anaphylaxis are caused by chemotherapy, iodinated radiocontrast media, NSAIDs, antibiotics, and NMBAs, among others [7]. Anaphylactic reactions to antibiotics are rare, partly because a preoperative AST is typically conducted. This test, which includes an intradermal test following a negative skin prick test, is considered a safe and reliable method for detecting immediate allergic hypersensitivity reactions [8]. Specifically, cephalosporins show a high negative predictive value; among patients with a history of cephalosporin use, the true positivity rate is only 1.4% out of 42.9%, indicating a low incidence of cefazolin anaphylaxis and the potential for false-positive results in routine screening, as previously reported [9]. In our case, the likelihood of cefazolin-induced anaphylaxis was low, given the negative result in the AST.

Anaphylaxis during the perioperative period occurs at a rate of approximately 1 in 10,000–20,000, representing a rare but potentially lethal complication related to anesthesia. Perioperative anaphylaxis usually manifests within minutes of anesthetic induction; the most common initial clinical features during severe reactions are pulselessness, desaturation, and difficult lung inflation due to severe bronchospasm [10]. In our case, only cardiovascular collapse was observed, which is an indication of severe anaphylaxis [11,12]; this met the criteria of the worst shock sign, as in previous report [13].

Opioid-induced anaphylaxis is very rare. Although morphine is known to release histamine, skin tests for phenylpiperidines such as alfentanil, fentanyl, sufentanil, remifentanil, and meperidine show that cross-reactivity is still uncommon [14]. However, there has been a report of anaphylaxis to fentanyl in a patient with a positive skin test for meperidine [5]. Thus, a marginal hypersensitivity to fentanyl cannot be conclusively attributed to remifentanil-related anaphylaxis, based on this previous report.

Propofol is an alkyl phenol in a lipid vehicle (soybean oil, egg lecithin, and glycerol). The incidence of anaphylactic reactions to propofol has been reported as 1 in 60,000 [1] and constitutes 1.2% of all anaphylactic reactions in France [14]. It has two allergenic components: the diisopropyl side chain and the phenol group. These are more commonly encountered in various environments: the isopropyl groups are in a wide range of skin care products, and the phenols are as components of mouthwashes, gargles, and throat lozenges. Most allergic reactions that develop after first exposure are due to sensitivity to the diisopropyl radical, whereas reactions upon re-exposure are typically due to the phenol group [15]. Because our case occurred after multiple exposures to propofol, it can be inferred that the phenol group acted as an epitope.

A reports of IgE-mediated anaphylaxis after multiple previous exposures has been documented [16]. Cases of anaphylaxis following re-exposure to propofol in patients without allergies to egg or soy have also been reported; these cases were characterized by anaphylactic shock after the third exposure and an immediate positive reaction to propofol in skin tests [17]. In contrast, our case involved the fourth exposure to the same drugs. Additionally, whereas most reactions occur rapidly within a few minutes, our event happened >30 min after exposure. In spite of the short context-sensitive half-life and clinical effect of propofol, this anaphylaxis was induced in an unexpectedly late active state and showed delayed symptoms unlike usual occurrence (within first 30 min) [18]. It involved only cardiovascular disturbances (decreased SpO_2_, hypotension, bradycardia leading to PEA) and no respiratory symptoms (no change in airway pressure and no bronchial spasm sound) or skin signs (erythema, edema, and urticaria).

Although these cardiovascular responses are not typical of anaphylactic reactions, prompt and appropriate cardiac arrest management aided in the treatment and recovery. When such shock symptoms occur, thorough evaluations are needed to determine the causative factors. Cardiac markers and mast cell serum tryptase testing are useful in acute differential diagnosis, and changes in EtCO_2_ waveform and airway pressure can be confirmed as having a pulmonary origin, including embolism. If feasible, TEE is recommended for the management of potential cardiac origin issues. In the ECG, TTE, and TEE of our case, the possibility of cardiogenic shock due to acute cardiac damage was low and not related to a disease of cardiac origin. Pulmonary embolism and acute coronary syndrome were also ruled out from these examinations.

Skin testing is the gold standard for the diagnosis of IgE-mediated reactions. Ideally, the test should be performed 4–6 weeks after the event to minimize the risk of a false-negative result. The skin prick test is less invasive and traumatic; thus, it was performed before the intradermal test, which is more sensitive [17].

Tryptase is recognized as an important marker for detection of acute anaphylaxis because it indicates mast cell degranulation. The increase in tryptase signifying anaphylaxis is more pronounced 1–2 h after the event, rather than immediately [19,20,21]. To increase reliability, ≥3 blood samples should be collected at different times and dates, consecutively [22]. This method allows for clearer confirmation of anaphylaxis through the timing of the rise in serum tryptase levels. Subsequently, skin testing can be used to identify the causative agent. In our case, three consecutive serum tryptase tests (immediately after the event, within ~2 h, and 8 h later) were performed, and the highest increase within ~2 h was suggestive of anaphylaxis. The skin prick test was negative, prompting the more sensitive intradermal test, which revealed a strong positive reaction to propofol, characterized by erythema and wheal. Through these findings, we predict that sensitization gradually increased with repeated exposure to propofol, and while propofol was the main contributor, other anesthetic drugs might have influenced the acceleration of this sensitization response. Unfortunately, the type of hypersensitivity could not be identified as histamine, IgE, or complement because these levels were not evaluated

Most cases of anaphylaxis occur acutely within 30 min [21], and 70% begin in <20 min [23]. It is rare for anaphylaxis to occur >45 min after exposure [13]. However, our event occurred >50 min later, beyond the usual occurrence time, and did not follow the common pattern of anaphylaxis. It is thought that this delayed response might be due to latent sensitization taking time to activate with repeated exposure to propofol, in the process developing into more fatal cardiovascular reactions than the typical anaphylactic reactions.

As previously mentioned, anaphylaxis due to propofol is rare. While most cases occur upon first exposure, our case atypically occurred during the fourth exposure. Although adverse reactions to propofol are common, critical damage is rare [1,4,24]. However, it is notable that in this instance, a severe anaphylactic reaction occurred during re-exposure to propofol in the subacute period. In addition, various sensitization examination (skin test, serum tryptase, histamine, immunoglobulins, complement, etc.) should be performed promptly and appropriately to prevent a recurrence.

## 4. Conclusions

Anaphylactic shock due to repeated exposures to propofol is uncommon. In patients with no previous history of allergies, multiple exposures to propofol can still precipitate severe anaphylactic reactions. It is important to remember that drugs previously tolerated can induce sensitization and potentially trigger anaphylaxis upon re-exposure. Therefore, medical professionals should be prepared to rapidly and appropriately respond through differential diagnosis when such reactions occur. Importantly, atypical anaphylaxis should not be dismissed, even if it does not present immediately or is not accompanied by typical skin symptoms and respiratory distress, such as bronchospasm.

## Figures and Tables

**Table 1:** Cardiac marker.

	POD0 (OR) ^1^	POD0 SICU ^2^	POD1	POD2
Troponin-T (TnT) (ng/mL)	0.005	0.059	0.047	0.031
NT-pro BNP (pg/mL)	51.6	39.8	197	229
CK-MB (ng/mL)	0.18	0.53	1.09	0.2

^1^: At the time of anaphylactic shock; ^2^: 2 h after anaphylactic shock, at the time of SICU admission; POD; post-operative day; NT-pro BNP: N-terminal pro b-type brain natriuretic peptide; CK-MB: creatinine kinase-MB.

**Table 2:** Serum tryptase levels.

	OR ^1^	PACU ^2^	SICU ^3^
Tryptase (ng/mL)	16.3	23.5	3.4

^1^; At the time of cardiac arrest; ^2^; 1 h after cardiac arrest; ^3^; 8 h after cardiac arrest; Reference range: 0~11.0; OR; operating room; PACU: post-anesthetic care unit, SICU; surgical intensive care unit.

**Table 3:** Drug sensitivity skin test results.

Drugs	Skin Prick Test			Intradermal Test			
	Dilution	Erythema *	Wheal	Dilution	Wheal (pre)	Wheal (post)	Erythema (post)
Esmeron ^1^	10 mg/mL	0 × 0	0 × 0	1:200	5 × 5	5 × 5	0 × 0
Fresofol MCT 1% ^2^	10 mg/mL	0 × 0	0 × 0	1:10	5 × 5	8 × 7	8 × 7
Fentanyl citrate	0.05 mg/mL	0 × 0	0 × 0	1:10	5 × 5	8 × 6	0 × 0
Histamine †		12 × 15	6 × 6	6 × 6	12 × 15	6 × 6	
Saline †		0 × 0	0 × 0	0 × 0	0 × 0	0 × 0	
Cefazolin ^3^				33 mg/mL	5 × 5	5 × 5	0 × 0
Cefazolin ^3^				3.3 mg/mL	5 × 5	5 × 5	0 × 0

^1^ Rocuronium 10 mg/mL; ^2^ propofol 10 mg/mL; ^3^ 1 g cefazolin; * size; † control.

## Data Availability

The data supporting the findings of this study are contained within the article.

## References

[1] Koul A., Jain R., Sood J. (2011). A critical incident report: Propofol triggered anaphylaxis. Indian J. Anaesth..

[2] Axon A.D., Hunter J.M. (2004). Editorial III: Anaphylaxis and anaesthesia—All clear now?. Br. J. Anaesth..

[3] Michavila Gomez A.V., Belver Gonzalez M.T., Alvarez N.C., Giner Muñoz M.T., Hernando Sastre V., Porto Arceo J.A., Induráin B.V. (2015). Perioperative anaphylactic reactions: Review and procedure protocol in paediatrics. Allergol. Immunopathol..

[4] Laxenaire M.C., Mata-Bermejo E., Moneret-Vautrin D.A., Gueant J.L. (1992). Life-threatening anaphylactoid reactions to propofol (Diprivan). Anesthesiology.

[5] Belso N., Kui R., Szegesdi I., Kakuja M., Kapitány K., Kemény L., Bata-Csörgo Z. (2011). Propofol and fentanyl induced perioperative anaphylaxis. Br. J. Anaesth..

[6] Brockow K., Wurpts G., Trautmann A., Pfützner W., Treudler R., Bircher A.J., Brehler R., Buhl T., Dickel H., Fuchs T. (2023). Guideline for allergological diagnosis of drug hypersensitivity reactions. Allergol. Sel..

[7] Park H.K., Kang M.G., Yang M.S., Jung J.W., Cho S.H., Kang H.R. (2017). Epidemiology of drug-induced anaphylaxis in a tertiary hospital in Korea. Allergol. Int..

[8] Lee S.H., Park H.W., Kim S.H., Chang Y.S., Kim S.S., Cho S.H., Min K.U., Kim Y.Y. (2010). The current practice of skin testing for antibiotics in Korean hospitals. Korean J. Intern. Med..

[9] Kwon J.W., Kim Y.J., Yang M.S., Song W.J., Kim S.H., Cho S.H., Chang Y.S. (2019). Results of Intradermal Skin Testing with Cefazolin according to a History of Hypersensitivity to Antibiotics. J. Korean Med. Sci..

[10] Dewachter P., Mouton-Faivre C., Emala C.W. (2009). Anaphylaxis and anesthesia: Controversies and new insights. Anesthesiology.

[11] Dewachter P., Mouton-Faivre C. (2008). What investigation after an anaphylactic reaction during anaesthesia?. Curr. Opin. Anaesthesiol..

[12] Harper N.J., Dixon T., Dugué P., Edgar D.M., Fay A., Gooi H.C., Herriot R., Hopkins P., Hunter J.M., Mirakian R. (2009). Suspected anaphylactic reactions associated with anaesthesia. Anaesthesia.

[13] Sugiyama Y., Takazawa T., Watanabe N., Bito K., Fujiyoshi T., Hamaguchi S., Haraguchi T., Horiuchi T., Kamiya Y., Maruyama N. (2023). The Japanese Epidemiologic Study for Perioperative Anaphylaxis, a prospective nationwide study: Clinical signs, severity, and therapeutic agents. Br. J. Anaesth..

[14] Hepner D.L., Castells M.C. (2003). Anaphylaxis during the perioperative period. Anesth. Analg..

[15] Saini B., Bandyopadhyay A., Kumar M., Agarwal S.M., Saini S. (2020). Propofol Induced Anaphylaxis, A Rare Anaesthetic Emergency: A Case Report. Int. J. Pharm. Sci. Rev. Res..

[16] Jenson R.D., Latham L.B., Vitalpur G.V., Dierdorf S.F. (2013). Immunoglobulin e-mediated anaphylaxis on the tenth exposure to cisatracurium in a 4-year-old child. A&A Case Rep..

[17] de Leon-Casasola O.A., Weiss A., Lema M.J. (1992). Anaphylaxis due to propofol. Anesthesiology.

[18] Manian D.V., Volcheck G.W. (2022). Perioperative Anaphylaxis: Evaluation and Management. Clin. Rev. Allergy Immunol..

[19] Vitte J., Sabato V., Tacquard C., Garvey L.H., Michel M., Mertes P.M., Ebo D.G., Schwartz L.B., Castells M.C. (2021). Use and Interpretation of Acute and Baseline Tryptase in Perioperative Hypersensitivity and Anaphylaxis. J. Allergy Clin. Immunol. Pract..

[20] Beck S.C., Wilding T., Buka R.J., Baretto R.L., Huissoon A.P., Krishna M.T. (2019). Biomarkers in Human Anaphylaxis: A Critical Appraisal of Current Evidence and Perspectives. Front. Immunol..

[21] Pitlick M.M., Volcheck G.W. (2022). Perioperative Anaphylaxis. Immunol. Allergy Clin. N. Am..

[22] Passia E., Jandus P. (2020). Using Baseline and Peak Serum Tryptase Levels to Diagnose Anaphylaxis: A Review. Clin. Rev. Allergy Immunol..

[23] Golden D.B. (2004). Patterns of anaphylaxis: Acute and late phase features of allergic reactions. Novartis Found Symp..

[24] Xuan G., Zhang Y., Cui J., Zhou J., Sui C. (2023). Propofol-associated serious adverse events: An analysis of the FAERS database. Biotechnol. Genet. Eng. Rev..

